# Research trends and hotspots evolution of artificial intelligence for cholangiocarcinoma over the past 10 years: a bibliometric analysis

**DOI:** 10.3389/fonc.2024.1454411

**Published:** 2025-02-13

**Authors:** Ke-xie Wang, Yu-ting Li, Sun-hu Yang, Feng Li

**Affiliations:** Department of General Surgery, Shanghai Traditional Chinese Medicine (TCM)-INTEGRATED Hospital of Shanghai University of Traditional Chinese Medicine, Shanghai, China

**Keywords:** cholangiocarcinoma, artificial intelligence, bibliometrics, convolutional neural networks, deep learning

## Abstract

**Objective:**

To analyze the research hotspots and potential of Artificial Intelligence (AI) in cholangiocarcinoma (CCA) through visualization.

**Methods:**

A comprehensive search of publications on the application of AI in CCA from January 1, 2014, to December 31, 2023, within the Web of Science Core Collection, was conducted, and citation information was extracted. CiteSpace 6.2.R6 was used for the visualization analysis of citation information.

**Results:**

A total of 736 publications were included in this study. Early research primarily focused on traditional treatment methods and care strategies for CCA, but since 2019, there has been a significant shift towards the development and optimization of AI algorithms and their application in early cancer diagnosis and treatment decision-making. China emerged as the country with the highest volume of publications, while Khon Kaen University in Thailand was the academic institution with the highest number of publications. A core group of authors involved in a dense network of international collaboration was identified. *HEPATOLOGY* was found to be the most influential journal in the field. The disciplinary development pattern in this domain exhibits the characteristic of multiple disciplines intersecting and integrating.

**Conclusion:**

The current research hotspots primarily revolve around three directions: AI in the diagnosis and classification of CCA, AI in the preoperative assessment of cancer metastasis risk in CCA, and AI in the prediction of postoperative recurrence in CCA. The complementarity and interdependence among different AI applications will facilitate future applications of AI in the CCA field.

## Introduction

Cholangiocarcinoma (CCA), a malignant neoplasm originating within the bile duct cells, presents a formidable challenge to medical intervention and is notably aggressive, leading to rapid mortality (1–3). This condition can manifest across all age groups, predominantly afflicting individuals over the age of 50, with an incidence that increases with age and is on the rise (4), accounting for 10%-25% of all hepatobiliary malignancies (5). Due to regional epidemiological factors, CCA exhibits a higher prevalence in Asia, with incidence rates in Shanghai, Korea, and Northern Thailand surpassing those of rare cancers (6–8). Surgical resection of the tumor remains the sole curative approach for CCA, applicable only to patients whose disease has not metastasized distantly; however, the vast majority of CCA cases are beyond the reach of surgical cure (9, 10). Studies indicate that up to 85% of CCA patients experience recurrence within three years post-surgery (11), and the five-year survival rate for those with inoperable tumors stands at 0% (12); the overall five-year survival rate for all CCA patients is merely 5% (13). The concealed location of the bile ducts complicates early detection of CCA, posing significant challenges for diagnosis and treatment. Currently, no specific blood test exists for the direct diagnosis of CCA, and surgical intervention to obtain tissue biopsies and accurately determine the disease stage is the only method, often at a point when the disease has advanced (14, 15). This underscores the urgent necessity for a method capable of early detection of CCA.

Artificial Intelligence (AI) encompasses a broad spectrum of technologies that have rapidly evolved, including machine learning (ML), deep learning (DL), and large models (LMs). Machine learning refers to the development of algorithms that can learn patterns from data and make predictions or decisions without being explicitly programmed. Traditional ML techniques, such as Support Vector Machines (SVMs) and Random Forests (RFs), have been widely applied in the medical field due to their ability to process structured data like laboratory results and clinical records. These algorithms are particularly useful for classification tasks, such as distinguishing between benign and malignant tumors, and for prognosis prediction based on predefined clinical parameters. In contrast, DL, a subset of machine learning, has made remarkable strides in recent years by employing neural networks with multiple layers. Deep learning algorithms, such as Convolutional Neural Networks (CNNs) and Recurrent Neural Networks (RNNs), excel at processing unstructured data like images and sequences, respectively. CNNs are highly effective in medical imaging tasks, such as identifying cholangiocarcinoma (CCA) from CT scans, MRI, or pathology slides, by recognizing intricate patterns that may not be visible to the human eye (16–19). RNNs, on the other hand, are valuable for analyzing sequential data, such as time-series patient health information, to track disease progression or predict treatment outcomes. Beyond traditional ML and DL, the emergence of large models (LMs), such as transformers, has introduced new possibilities in AI. These models, built on architectures like Generative Pre-trained Transformers (GPT) and Vision Transformers (ViTs), can process vast amounts of multimodal data, such as combining textual health records with medical imaging. Large models are characterized by their scalability and ability to learn from diverse, complex data, making them especially useful in scenarios requiring high-dimensional data integration and cross-domain analysis. In the field of CCA, large models are being explored for their potential to enhance the precision of diagnostic and prognostic predictions, providing clinicians with more comprehensive insights. By leveraging these diverse AI technologies—ML, DL, and LMs—researchers and clinicians can analyze extensive patient health data to predict survival rates and responsiveness to specific treatment plans, thereby enabling the development of personalized treatment strategies for CCA patients (20). With the rapid development of AI technology and the continuous emergence of novel applications in healthcare, there is an urgent need for timely and effective literature data analysis in this field, which could significantly enhance researchers’ understanding and guide subsequent studies (21–23).

Currently, research on the types of literature concerning AI in CCA is scarce, with only a few reviews and one meta-analysis published (24–27). These studies provide a commendable summary of the current state and future directions of AI applications in CCA, especially in preoperative imaging diagnostics and evaluation (28). However, they fail to offer insights into the field’s evolving trends over time or detailed citation information behind the publications, such as author collaboration networks, geographical development relationships, and core keyword information. In contrast, bibliometric analysis (BA), utilizing the theories and methodologies of bibliometrics and informatics, offers a more profound, insightful perspective, objectively and visually presenting the quantity, quality, and impact of literature (29, 30). Therefore, the timely and effective application of BA to analyze current hotspots in AI research on CCA holds significant value.

This study aims to objectively and visually summarize the publication data on AI applications in the CCA domain through BA, including trends in annual publication numbers, core keyword clustering, authors, countries, institutions, and journal citation information, and to distill the research hotspots and future challenges faced by the field.

## Materials and methods

### Data sources

The data for this study were derived from the Web of Science Core Collection (WoSCC). This database is not only a vital resource for researchers worldwide for literature retrieval, research evaluation, and information exchange, but its unique advantage lies in the inclusion of detailed citation index information behind publications, which can be directly utilized for BA (31, 32). Consequently, almost all BAs currently employ the WoSCC. For ease of comparison and validation with similar studies, this database was also chosen as the source of publication data for this research.

### Search strategy

**Figure 1:**
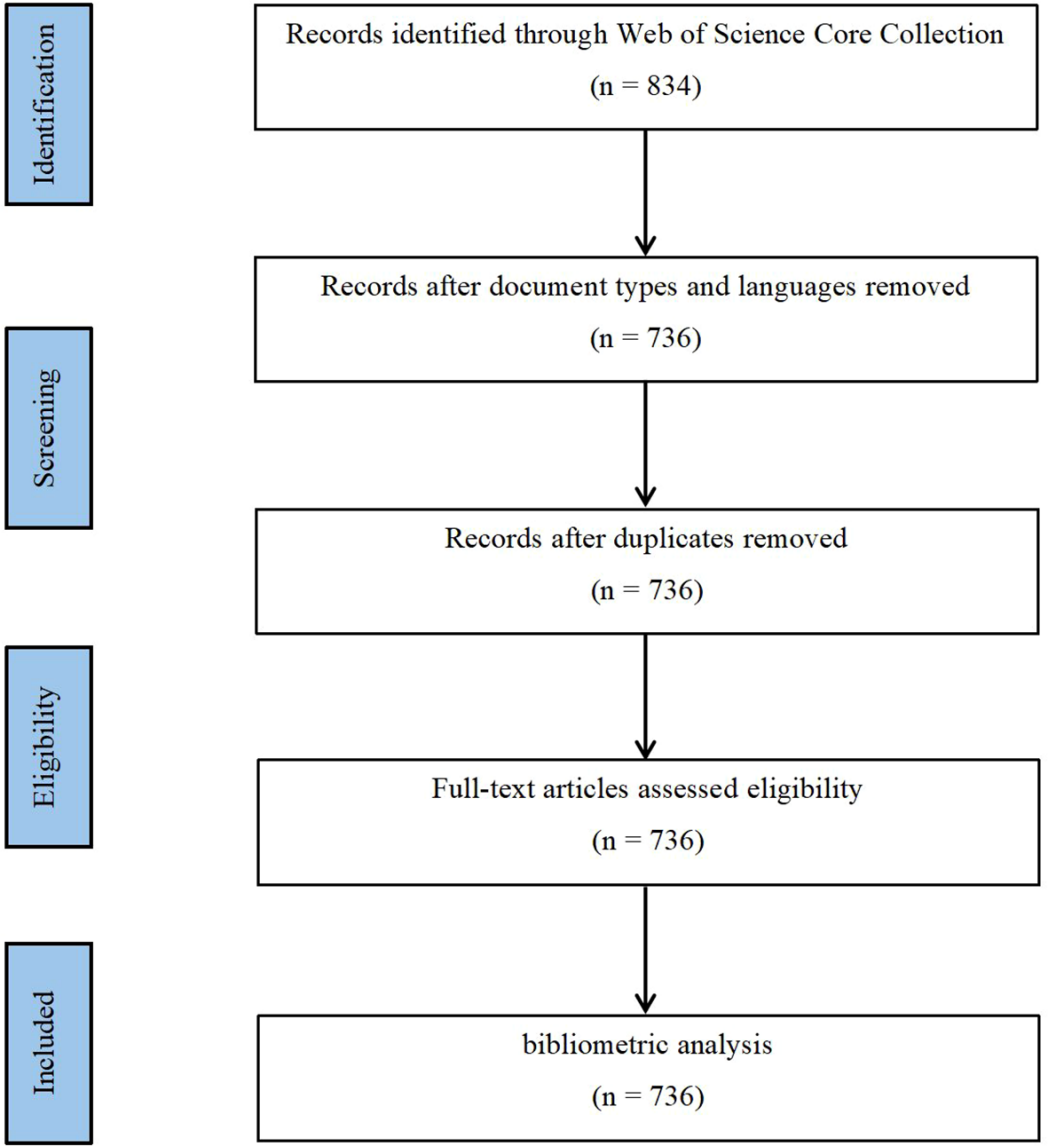
Map of literature screening process related to AI for CCA.

The research strategy for this study was crafted around search terms related to “Artificial Intelligence” and “Cholangiocarcinoma,” resulting in a comprehensive set of search terms (**Supplementary Table 1**). The search spanned from January 1, 2014, to December 31, 2023, covering a complete decade to ensure the thematic integrity of the study’s focus on “the past 10 years.” To maintain consistency with similar research methodologies, facilitating comparison and validation, there were no restrictions on the country of publication, while the language was specified as “English” and the type of study as “article.” The process for selecting publications is depicted in **Figure 1**. Ultimately, this study included 736 publications that met the criteria.

### Analysis tool

This study employed CiteSpace 6.2.R6, developed by Professor Chaomei Chen of Drexel University. CiteSpace software is a visualization tool that, compared to other BA research software, is distinguished by its ability to identify research hotspots and frontier fields as well as to forecast the future development trends of specific areas. This makes it one of the most popular tools in the field of BA. The process of conducting a BA with CiteSpace typically involves data collection, utilization of CiteSpace, data analysis, and interpretation of the analysis results. Specifically, we exported the search results in the “download.txt” format supported by CiteSpace, choosing the “full record and cited references” data parameter to ensure that the exported data included citation information, abstracts, keywords, and all other materials. After deduplication and verification of the data, we set node types (such as keywords, authors, institutions, etc.) and thresholds.

To enhance the clarity of the final visualization and facilitate the observation of relationships between publications, we set the time slice to “1 year.” (33) This allows us to capture annual changes in the literature, providing a detailed view of the evolving research trends in AI applications within the CCA field. The one-year interval was chosen to balance granularity with manageability, ensuring that data from each year could be analyzed without creating overly fragmented or sparse datasets. Additionally, we used the g-index to identify influential publications in each time slice, with a k value set to 50 (34). This means that the top 50 most-cited papers were selected for analysis in each year. The choice of 50 as the threshold was based on an examination of citation patterns within our dataset, which showed that this number adequately captures the key publications while maintaining a manageable network size for visualization. To simplify and optimize the generated network maps, we applied the Pathfinder algorithm (35). This algorithm reduces network complexity by removing redundant connections, allowing us to focus on the most significant relationships between publications. By doing so, we ensured that the network maps highlighted the most meaningful nodes and paths, making it easier to identify research hotspots and key trends within the field. Finally, we identified and analyzed clusters within the network, interpreting key nodes and paths to reveal the primary research topics and their evolution over time.

## Results

### Analysis of annual publications and trends

**Figure 2:**
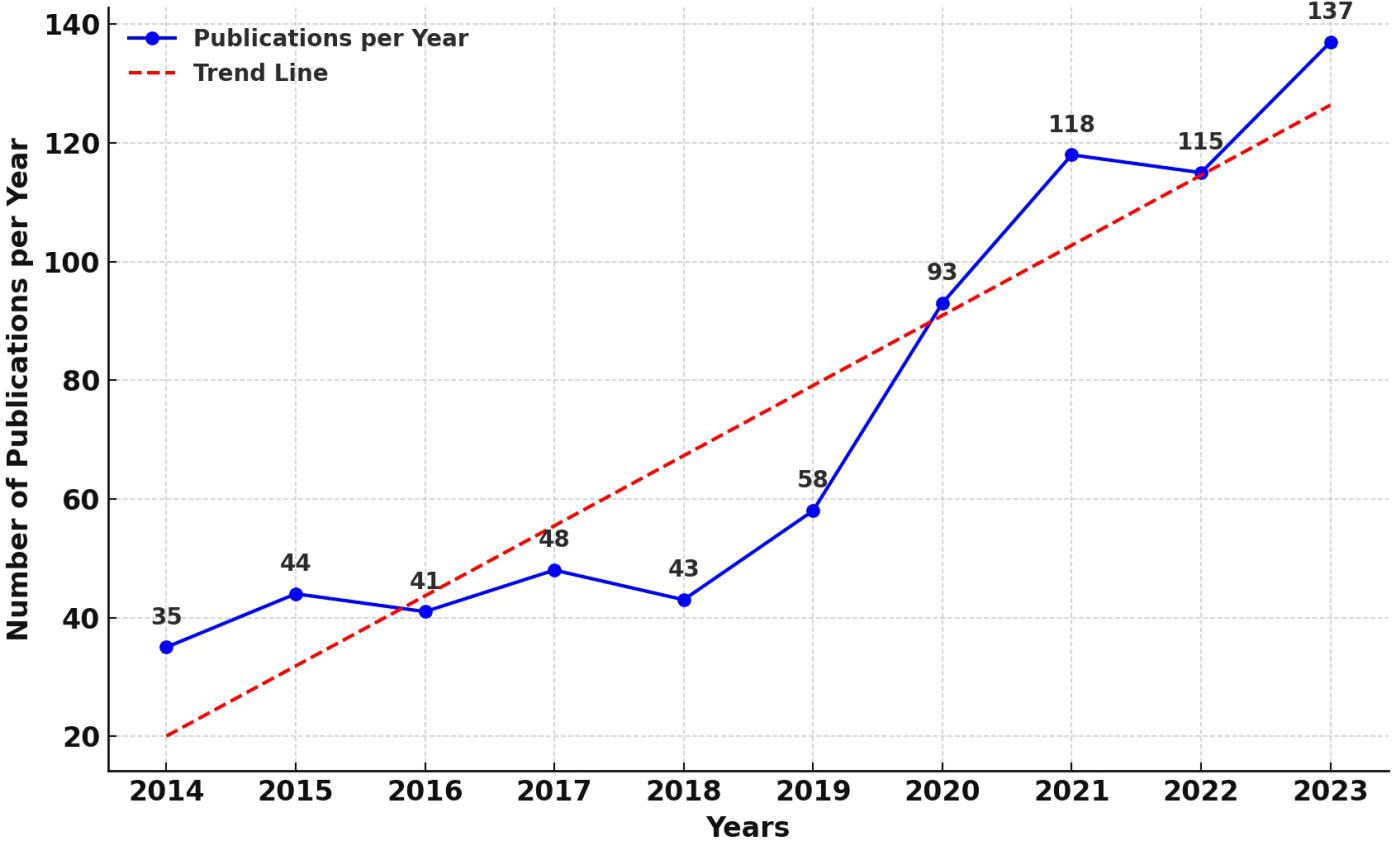
Map of annual publications related to AI for CCA.

**Figure 2** presents a line graph illustrating the annual publication count in the field of AI applications to CCA. An analysis reveals a bifurcated trend in publication volume: the first phase, spanning from 2014 to 2019, exhibits slow growth, indicating that the application of AI in the medical field was in its nascent and experimental stages; the second phase, from 2019 to the present, shows a rapid increase in publication numbers, signifying the medical community’s recognition of the value of AI applications in CCA, leading to its widespread adoption. Additionally, for ease of observation of the trend, a trend line has been added to the graph. It is observed that the publications in the domain of AI applications to CCA are on a rapid growth trajectory, with the volume of publications likely reaching new peaks in the coming years.

### Analysis of keywords

**Figure 3:**
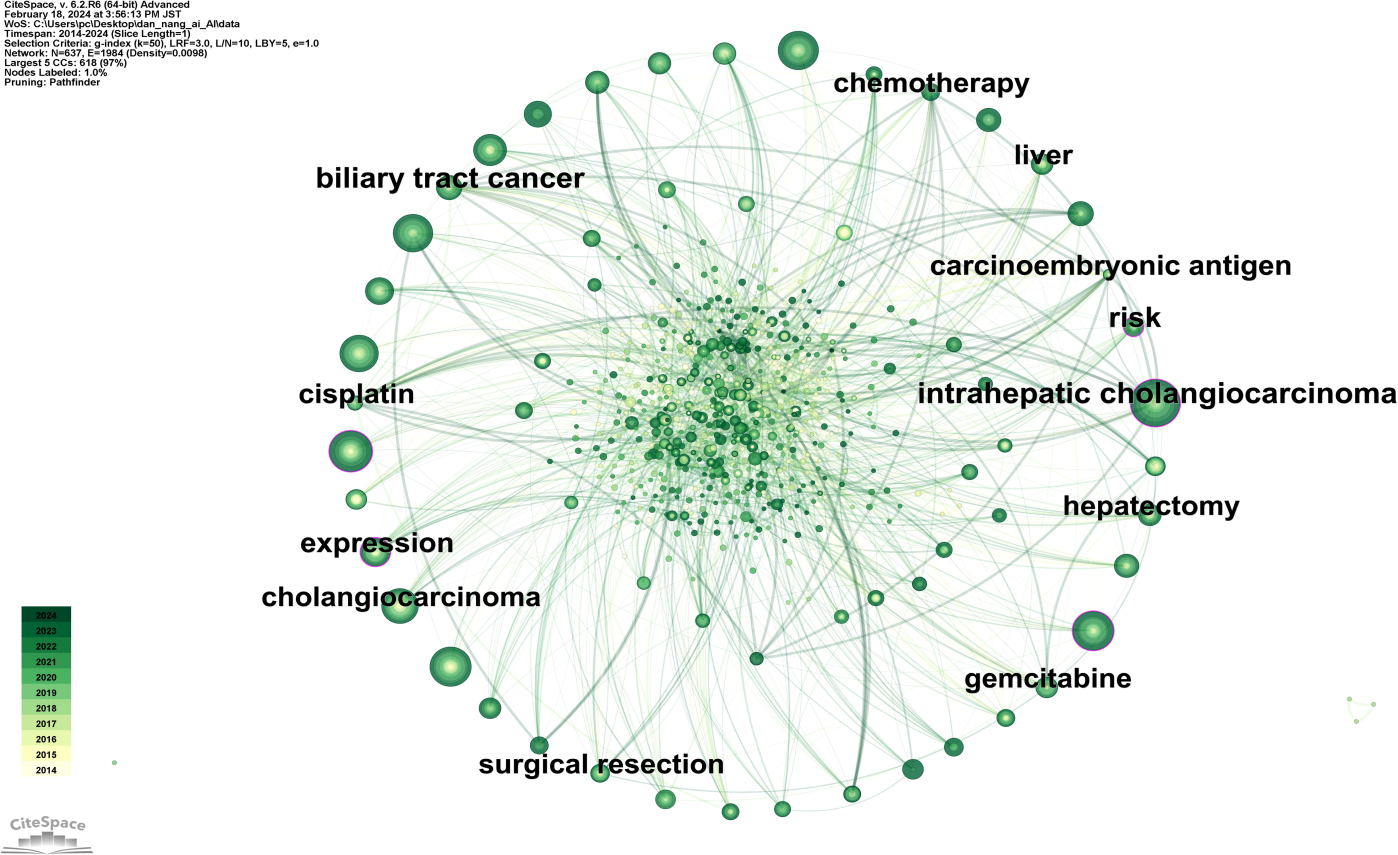
Map of keywords related to AI for CCA.

**Figure 3** showcases a keyword network diagram composed of 637 keywords connected by 1984 links. The top 10 most frequently occurring keywords are listed in **Supplementary Table 2**, in descending order: intrahepatic cholangiocarcinoma (141 occurrences), hepatocellular carcinoma (105 occurrences), cancer (99 occurrences), diagnosis (97 occurrences), survival (92 occurrences), resection (91 occurrences), management (90 occurrences), cholangiocarcinoma (85 occurrences), hilar cholangiocarcinoma (66 occurrences), carcinoma (58 occurrences). Among these, intrahepatic cholangiocarcinoma (0.12), expression (0.12), diagnosis (0.11), cancer (0.10), and risk (0.10) have higher centrality (indicated by purple rings).

**Figure 4:**
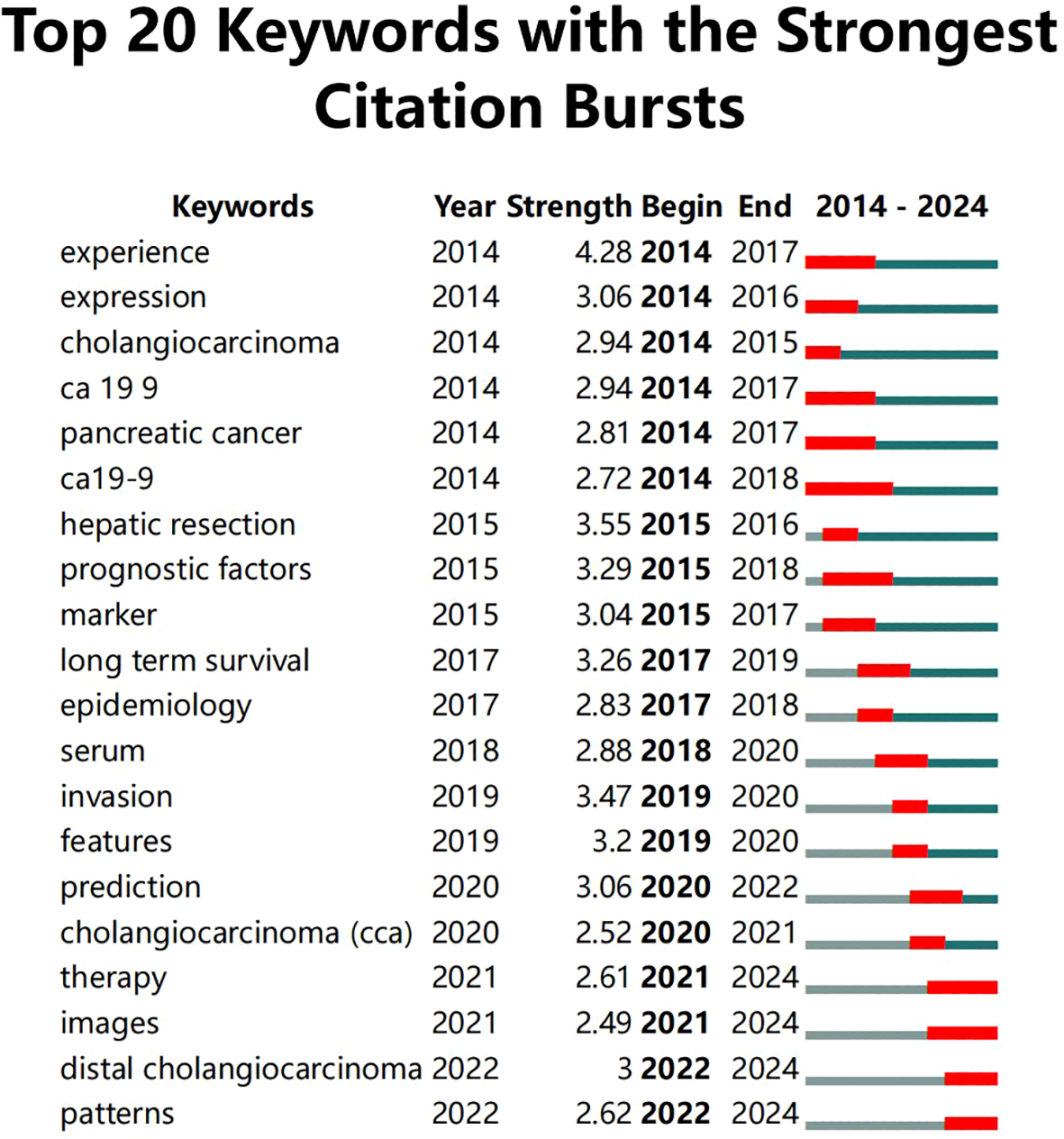
Map of keywords timeline related to AI for CCA.

**Figure 4** illustrates a timeline graph depicting the development of these keywords over time. Through cluster analysis, six core clusters were identified: Cluster #0 focusing on AI methods such as deep learning and machine learning; Cluster #1 on carcinoembryonic antigen and tumor markers; Cluster #2 on liver resection and laparoscopic hepatectomy; Cluster #3 on cancer types such as perihilar cholangiocarcinoma and left-sided gallbladder; Cluster #4 on liver-related issues such as intrahepatic cholangiocarcinoma and liver neoplasms; Cluster #5 on imaging diagnostic markers like endoscopic retrograde cholangiography and positron emission tomography.

**Figure 5:**
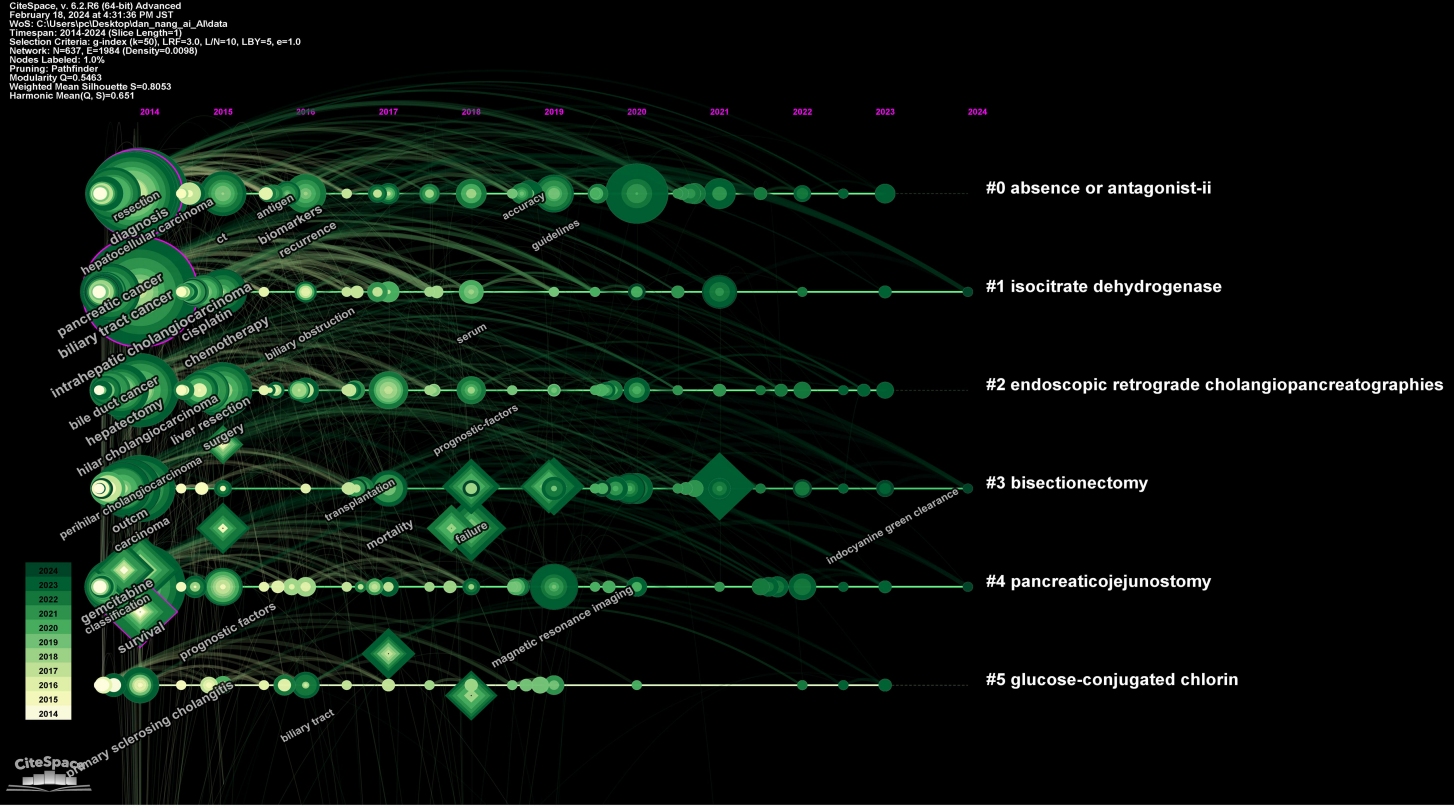
Top 10 keywords with the strongest citation bursts.

Moreover, information on keywords with the Strongest Citation Bursts was extracted (**Figure 5**), revealing that from 2014 to 2019, clinical treatments and care protocols for CCA received significant attention. Post-2019, the focus shifted towards the application of artificial intelligence algorithms and the diagnosis of cancer. It was also noted that although “ca19-9” had the longest burst duration (5 years), it only continued until 2022, after which the application of AI in CCA diagnosis became predominant.

### Analysis of authors

**Figure 6:**
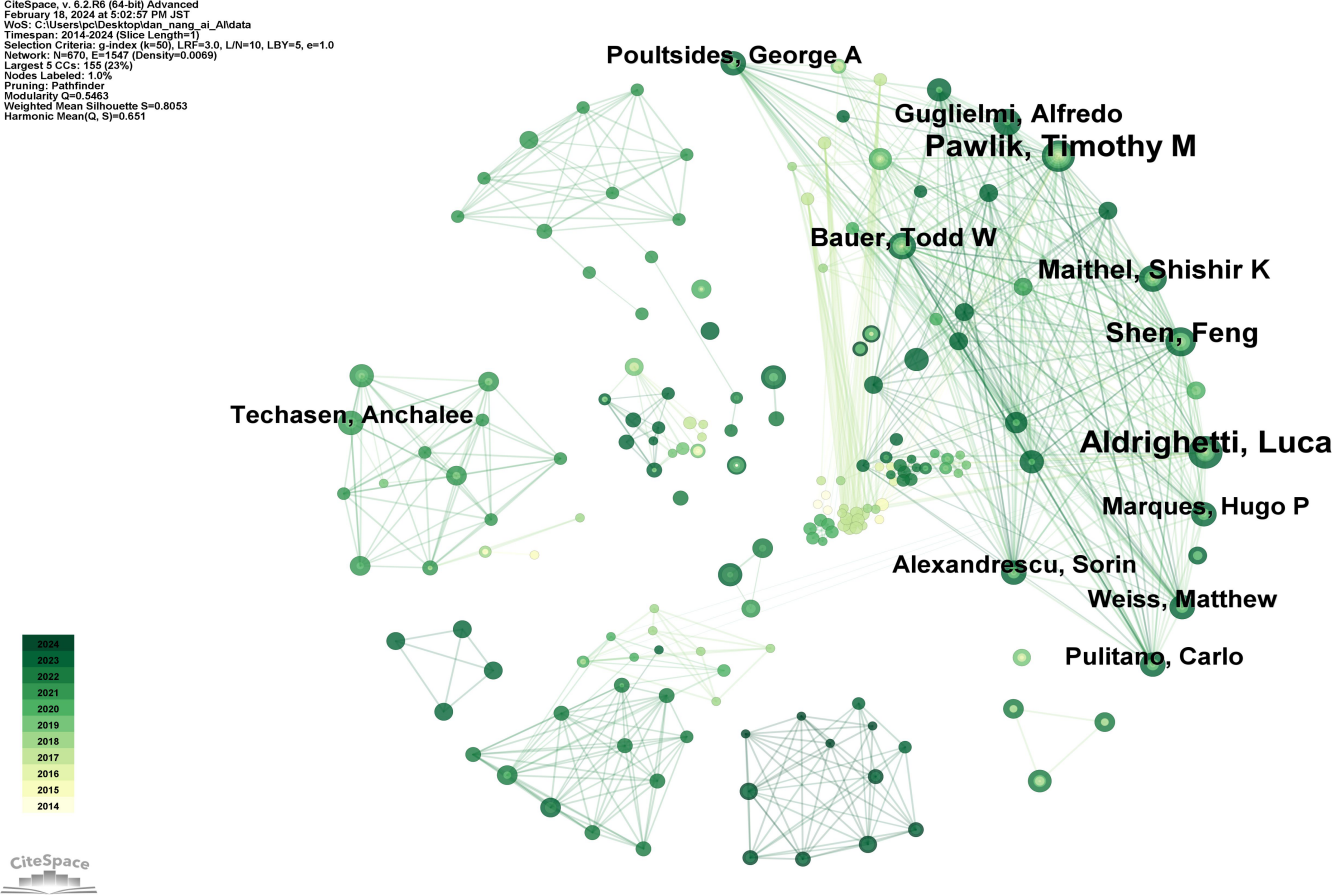
Map of authors related to AI for CCA.

**Figure 6** reveals that in the domain of AI applications to CCA, a total of 670 authors have contributed to publications, forming a collaboration network with 1547 interconnecting lines. The top 10 authors by publication count are listed in **Supplementary Table 3**, in descending order: Aldrighetti, Luca (16 publications), Pawlik, Timothy M (15 publications), Shen, Feng (11 publications), Maithel, Shishir K (10 publications), Guglielmi, Alfredo (9 publications), Bauer, Todd W (9 publications), Poultsides, George A (8 publications), Weiss, Matthew (8 publications), Alexandrescu, Sorin (8 publications), and Pulitano, Carlo (8 publications). It is observed that although the top 10 authors hail from diverse countries, they share a dense network of collaboration. This indicates that a stable core group of authors has been established in the field of AI applications to CCA, and these collaborative relationships are highly beneficial for large-sample, multi-center research and studies involving data sharing. For example, Aldrighetti (36) published an international multi-center study demonstrating the safety and efficacy of staged liver resection. Bagante (37) utilized classical survival models and classification and regression tree models to analyze 1116 patients from 14 international centers, finding a strong non-linear correlation between the size and number of intrahepatic CCA tumors and survival rates following resection of intrahepatic CCA.

### Analysis of countries and institutions

**Figure 7:**
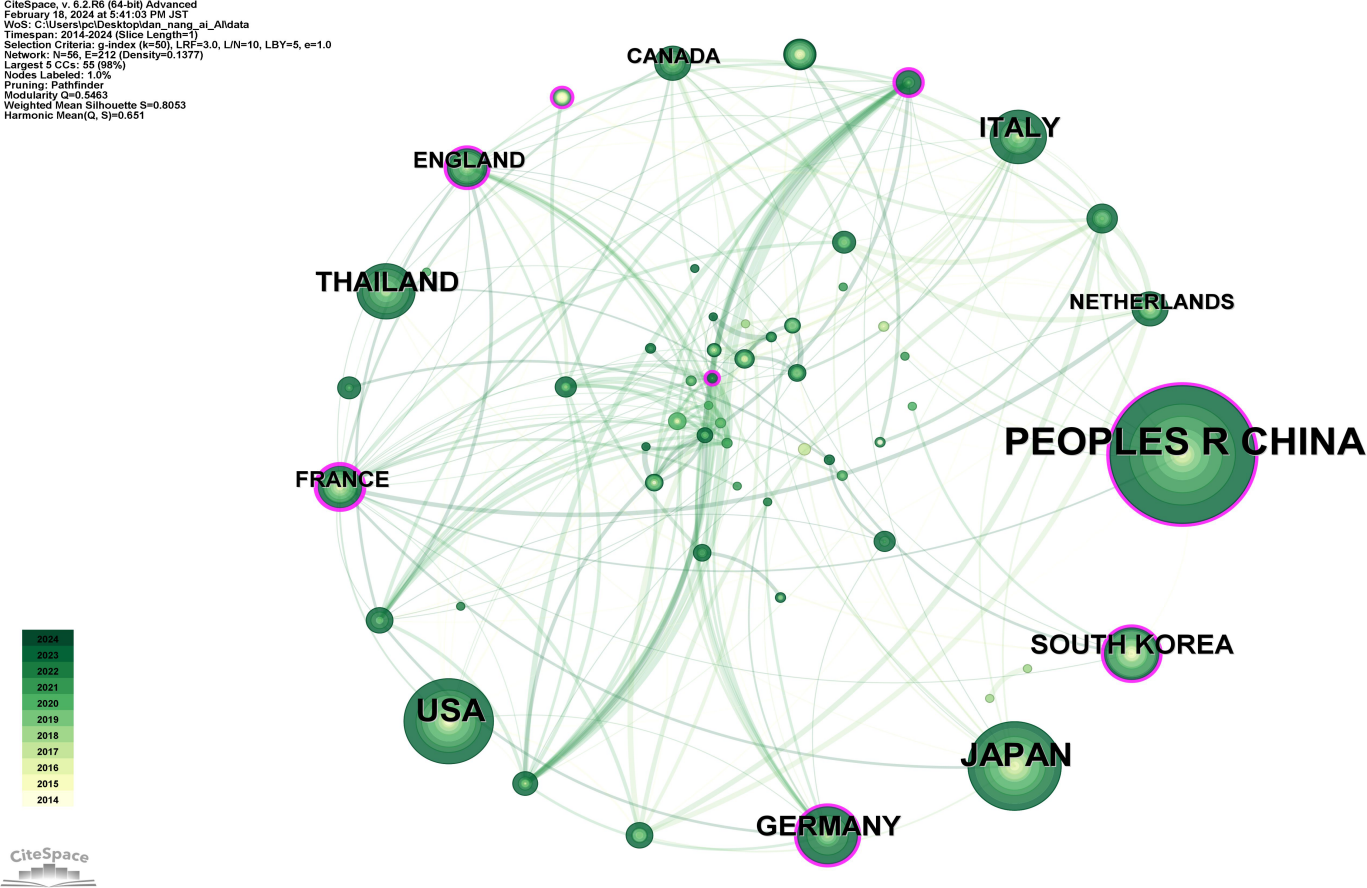
Map of countries related to AI for CCA.

**Figure 7** depicts a network diagram of international collaborations involving 56 countries and 212 collaboration links. **Supplementary Table 4** lists the top 10 countries by publication count, in descending order: PEOPLE’S REPUBLIC OF CHINA (292 publications), JAPAN (128 publications), USA (116 publications), THAILAND (57 publications), GERMANY (55 publications), ITALY (52 publications), SOUTH KOREA (48 publications), FRANCE (24 publications), ENGLAND (22 publications), CANADA (20 publications). This reveals that China has the highest volume of publications in the domain of AI applications to Cholangiocarcinoma (CCA), exceeding Japan, the second highest, by 128.13%, thus underscoring China’s pivotal influence in this field. Additionally, France is noted for its highest centrality, closely associated with a high-level clinical study published jointly by 17 French institutions, reflecting the significant collaborative impact within the country (38).

**Figure 8:**
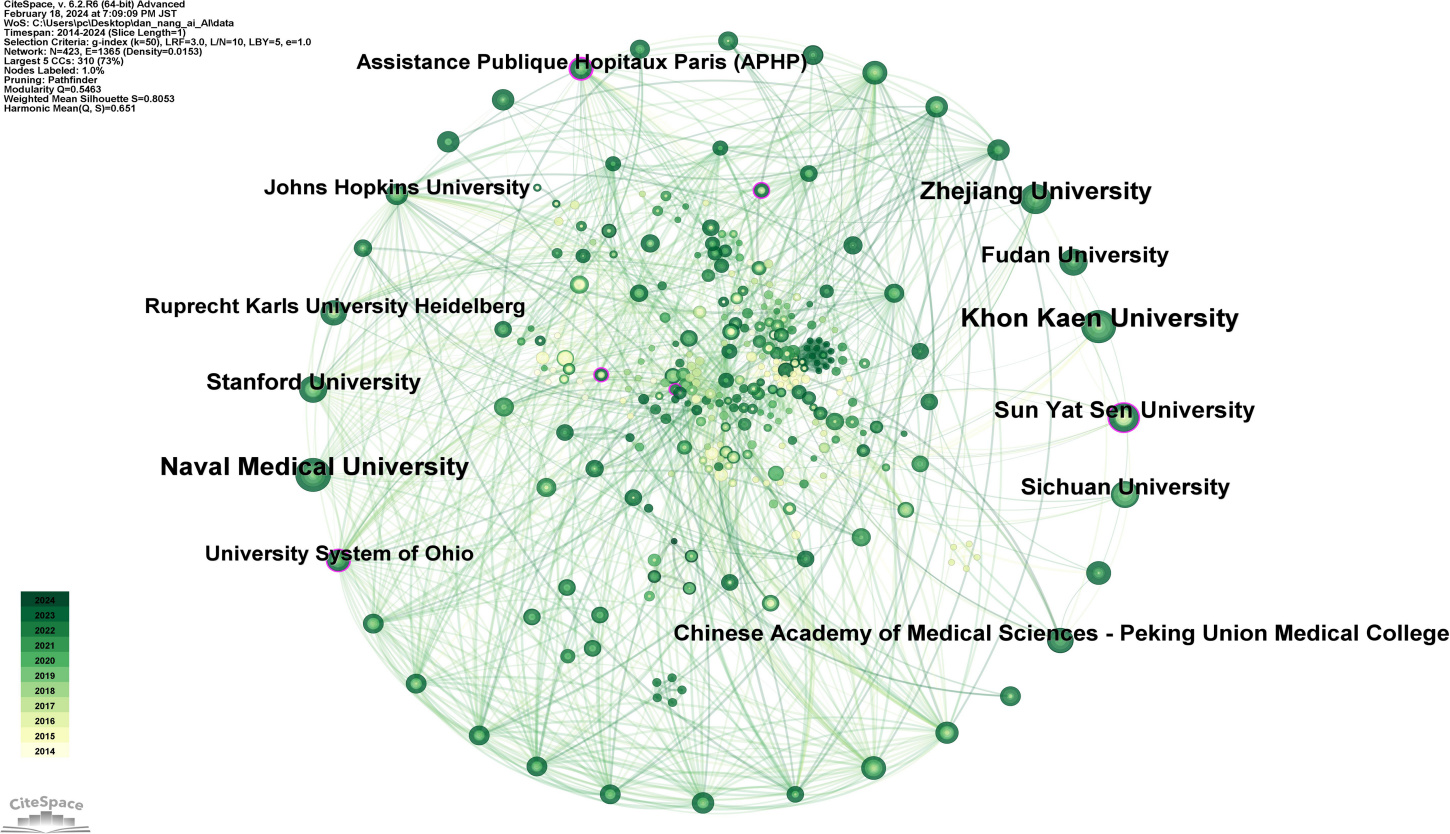
Map of institutions related to AI for CCA.

Moreover, an analysis visualizing the academic institutions corresponding to these countries (**Figure 8**) lists the top 10 institutions by publication count in **Supplementary Table 5**, in descending order: Khon Kaen University (37 publications), Naval Medical University (34 publications), Zhejiang University (29 publications), Sun Yat-Sen University (23 publications), Sichuan University (22 publications), Stanford University (22 publications), Chinese Academy of Medical Sciences - Peking Union Medical College (21 publications), Fudan University (21 publications), Assistance Publique Hopitaux Paris (APHP) (17 publications), Johns Hopkins University (17 publications). With the highest number of publications, Khon Kaen University, a prestigious institution in Thailand, signifies that Thailand has reached a leading international position in the application of AI to CCA.

### Analysis of cited journals

**Figure 9:**
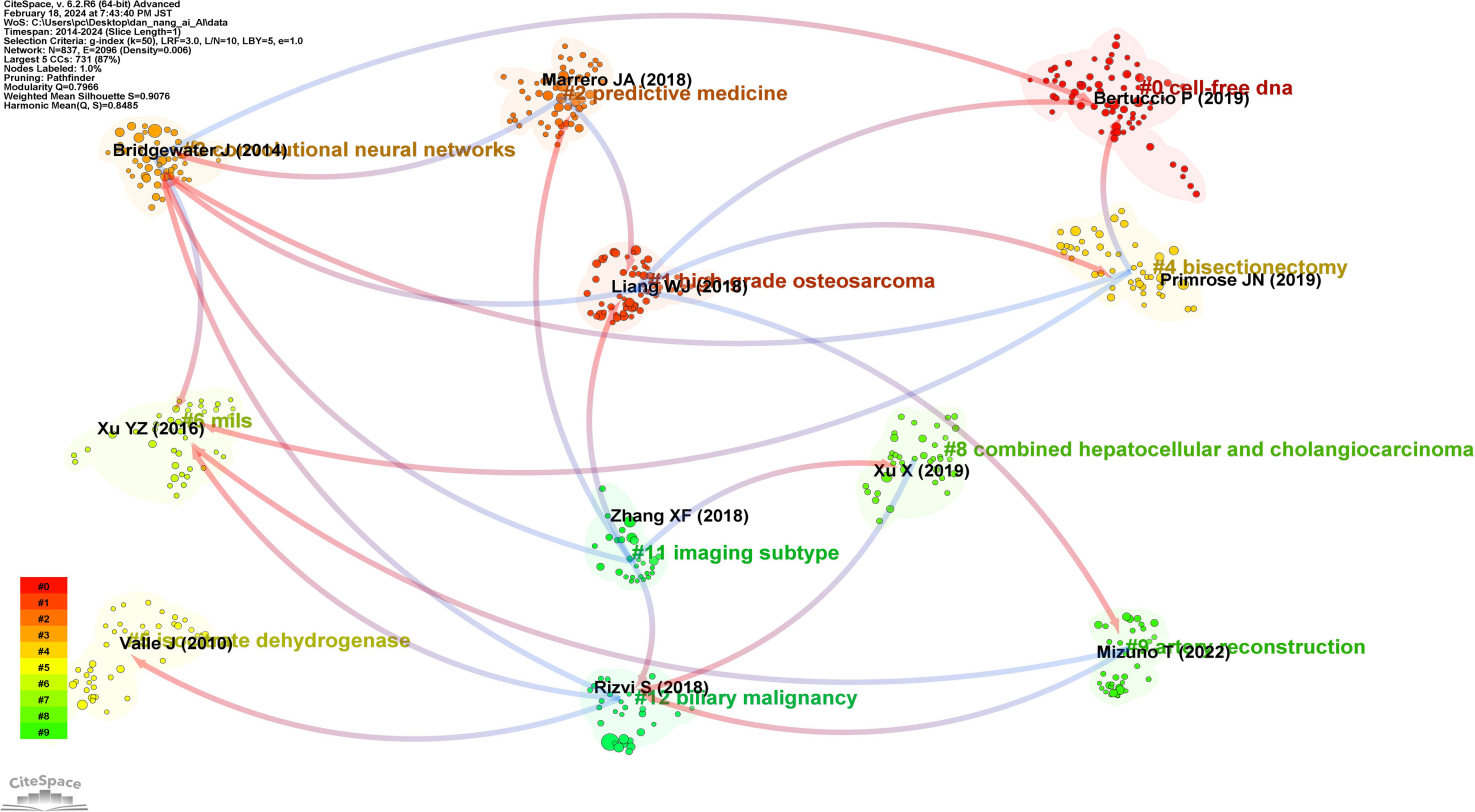
Map of cited journals related to AI for CCA.

**Figure 9** shows a network diagram of cited journals consisting of 835 journals and 4263 connections. The top 10 journals by citation count are listed in **Supplementary Table 6**, in descending order: *HEPATOLOGY* (292 citations), *ANN SURG* (285 citations), *ANN SURG ONCOL* (274 citations), *J HEPATOL* (252 citations), *HPB* (208 citations), *SURGERY* (192 citations), *J GASTROINTEST SURG* (190 citations), *J CLIN ONCOL* (187 citations), *WORLD J GASTROENTERO* (186 citations), *WORLD J SURG* (180 citations). Among these, *HEPATOLOGY*, with an impact factor of 14, stands as the most influential journal in this field.

### Analysis of references

**Figure 10:**
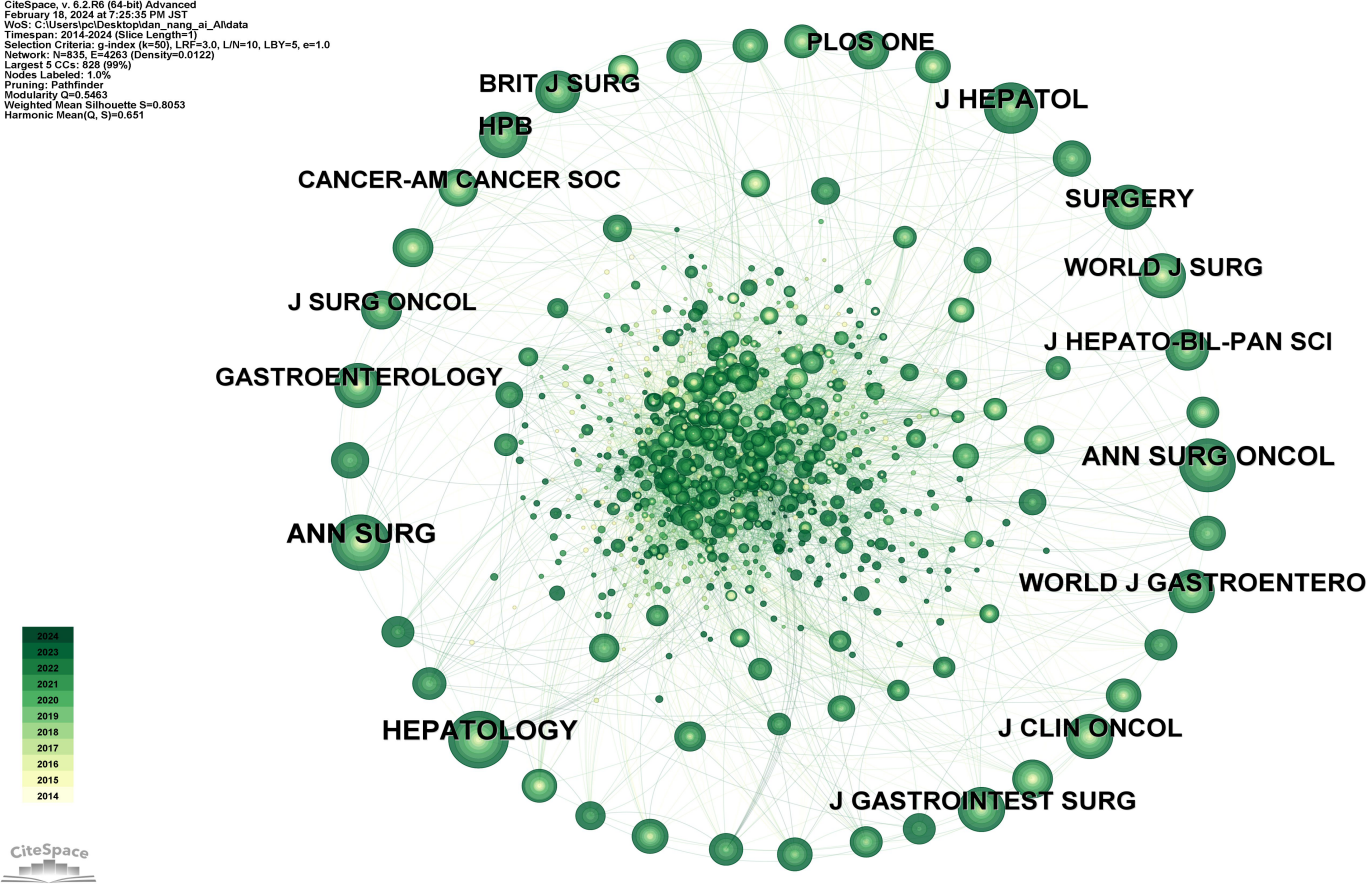
Map of reference of cluster dependencies related to AI for CCA.

**Figure 10** is a disciplinary development relationship diagram derived from references. Arrows indicate the developmental relationship between different disciplines, with the arrowhead pointing towards the latest knowledge frontiers and the tail representing the origins in foundational literature. It was observed that the network of disciplinary development in the field of AI applications to CCA is quite rich, showcasing the characteristic of multiple disciplines intersecting and integrating. This interdisciplinarity is conducive to the production of high-level evidence in the field.

## Discussion

### General information

In this study, we adopted a meticulous and holistic approach to delve into the application and developmental trajectory of AI technology in the field of CCA research from 2014 to 2023. Through extensive searches and analyses of the WoSCC database, we not only aggregated a vast array of publication data but also employed advanced data visualization techniques to objectively reveal the deep-seated changes in knowledge structures and developmental trends within this academic field.

Our findings highlighted two distinct phases of growth: a steady increase during the initial phase (2014 to 2019) and an accelerated growth in the subsequent phase (2019 to present). Notably, the year 2019 marked a significant turning point in the application of AI technology in CCA research, foreshadowing a qualitative leap in research outputs in the forthcoming years.

By analyzing the keyword network and temporal sequences, we traced the evolution of research focuses and observed a diversification of research themes. Early research concentrated on traditional treatment methods and care strategies for CCA, while post-2019, there was a significant pivot towards the development and optimization of AI algorithms, and their application in early cancer diagnosis and treatment decision-making, reflecting the profound impact of technological advancement on research interests.

Additionally, our study uncovered the pivotal role of transnational scientific collaboration in advancing AI application research in the CCA field through the analysis of top authors and their collaboration networks. This international cooperation not only facilitated the conduct of large-scale, multi-center studies but was also crucial for promoting data sharing, knowledge dissemination, and the application of innovative methods. Case studies demonstrated that cross-national collaborative research not only elevated the quality and impact of the studies but also provided empirical foundations for exploring more complex treatment methodologies.

Furthermore, our analysis paid special attention to the impact of geographical distribution. China’s leading volume of research publications in the application of AI to CCA underscored its significant position in the global scientific research landscape. Meanwhile, France’s highest centrality reflected its leading position in conducting high-quality, multicentric research through national and international collaboration. The outstanding performance of Thailand’s Khon Kaen University highlighted Thailand’s international influence in this research domain.

Moreover, by analyzing the journals with the highest impact factors, we underscored the role of high-impact journals in disseminating top-tier research findings. An in-depth analysis of references revealed the interdisciplinary integration trend in AI CCA research, showcasing the interaction and fusion of biomedical science, computer science, data analysis, and other fields, providing new perspectives and methodologies for CCA research and treatment.

On a creative expansion, we further envisioned the potential applications of AI technology in future CCA research, including optimizing personalized treatment plans through deep learning algorithms, utilizing big data analysis to predict disease progression trends, and developing novel non-invasive early diagnostic techniques. These exploratory visions are not only based on current research trends but also reflect the broad prospects of AI technology in the future healthcare field.

In conclusion, this study not only unveiled the developmental trajectory and trend changes of AI in the CCA research field but also highlighted the key role of interdisciplinary research and international collaboration in driving innovation in this domain. With the continuous advancement of AI technology and the deepening of global scientific cooperation, there is reason to anticipate that AI will play an increasingly important role in the diagnosis, treatment, and prevention of cholangiocarcinoma, bringing more hope and possibilities to patients.

### Research hotspots

Through the summarization of citation information and their clustering relationships, we identified three main research hotspots in the application of AI to CCA: AI in the diagnosis and classification of CCA, AI in the preoperative assessment of cancer metastasis risk in CCA, and AI in the prediction of postoperative recurrence in CCA.

#### AI in the diagnosis and classification of CCA

In the domain of CCA diagnosis and classification, the application of AI technology, particularly deep learning, has significantly enhanced the accuracy of identifying pathological features from medical images. A series of studies (19, 23, 38–40) have demonstrated that deep learning models can discern subtle differences in medical image analysis, achieving diagnostic accuracy comparable to, and sometimes surpassing, that of pathologists. Moreover, various AI algorithms have undergone different validations. For instance, Sun (41) demonstrated the accuracy of AI in CCA diagnosis using CNNs to analyze patients’ pathological datasets. Their team also proposed an unsupervised learning method for segmenting histopathological images based on incomplete labels, which may provide new insights into improving diagnostic accuracy for cholangiocarcinoma (42). Moreover, Chakrabarti (43) developed a lightweight neural network that achieved validation accuracy, precision, and recall rates of 98.40%, 100%, and 100%, respectively. Logeswaran (44) developed an automatic preliminary detection system using a multilayer perceptron, obtaining 88% in test results. Bagante (45) achieved an accuracy rate of 82% using artificial neural networks. Gao (46) and their team created a user-friendly AI-based predictive platform for CCA to facilitate researchers’ use.

#### AI in preoperative cancer metastasis risk assessment in CCA

AI models can integrate multi-source data, including patient imaging data, biomarkers, and genetic information, to identify patients at high risk of metastasis, aiding physicians in devising personalized treatment plans. This includes selecting the most suitable surgical method, radiotherapy, chemotherapy, or other treatment strategies. In existing research, Gao (47) applied CNNs to predict microvascular invasion in CCA, achieving an accuracy of 86.8%. Liu (48) improved the preoperative risk assessment of CCA using (SVMs. Huang (49) used Random Forest to predict ICC lymph node metastasis, enhancing prediction accuracy. Additionally, an online calculator was developed using a database from multiple centers for calculating death risk and overall survival, benefiting disease staging and prognosis analysis (50).

#### AI in the prediction of postoperative recurrence in CCA

AI algorithms can analyze postoperative clinical data and follow-up imaging materials of patients to identify early signs of recurrence, thus enabling the early prediction of recurrence. This not only helps in formulating personalized monitoring plans for CCA patients but also aids doctors in devising more personalized subsequent treatment plans, including secondary surgery, adjuvant chemotherapy, or targeted therapy. For example, Song (51) applied the LightGBM model to predict the early postoperative recurrence risk of CCA, with a sensitivity of 94.6%. Bo (52) utilized patients’ radiomic feature curves, calibration curves, and decision curves to improve the predictive performance of early postoperative intrahepatic cholangiocarcinoma recurrence. Chen (53) used six algorithms: logistic regression, RF, neural networks, Bayesian, SVM, and Extreme Gradient Boosting (XGBoost), to effectively predict early recurrence of intrahepatic cholangiocarcinoma after curative liver resection. Wakiya (54) applied a residual convolutional neural network for the early postoperative recurrence prediction of intrahepatic cholangiocarcinoma, with sensitivity, specificity, and accuracy rates of 97.8%, 94.0%, and 96.5%, respectively.

## Future research directions

While significant progress has been made in applying AI to CCA diagnosis, risk assessment, and postoperative recurrence prediction, several potential research directions remain underexplored: (1) Multimodal Data Integration: Future research can enhance AI models by incorporating genomics, proteomics, and clinical data, improving predictive accuracy and patient stratification. (2) Model Interpretability: Developing explainable AI models to improve clinical trust and adoption is essential for future applications. (3) Generalizability and Robustness: Larger, diverse datasets and external validations are needed to ensure models work across different populations and clinical settings. (4) Real-Time AI Applications: Exploring real-time AI integration into clinical workflows, such as intraoperative decision-making and postoperative monitoring, offers promising improvements. (5) Personalized Medicine: Adaptive AI systems that learn from evolving patient data could enhance personalized treatment for CCA over time. (6) Ethical and Regulatory Considerations: Addressing AI-related ethical, privacy, and regulatory challenges is critical for clinical implementation and widespread adoption.

### Clinical value and innovation of AI in CCA management

The clinical value of applying AI in CCA management lies in its ability to enhance diagnostic precision, risk assessment, and personalized treatment. AI models, particularly deep learning algorithms, improve the accuracy of CCA diagnosis by identifying subtle pathological features, reducing diagnostic errors, and enabling earlier interventions. Moreover, AI-driven preoperative risk assessments provide a comprehensive understanding of patient profiles, supporting more tailored surgical and therapeutic decisions.

This study’s innovation is demonstrated through the integration of multi-source data—imaging, genomics, and clinical histories—into AI models, creating a more holistic approach to patient management. Additionally, AI’s role in predicting postoperative recurrence introduces a proactive element to patient care, facilitating early detection and timely treatment adjustments. The potential for real-time AI integration into clinical workflows, offering adaptive and personalized treatment plans, further highlights the innovative clinical impact of this research.

## Conclusion

The comprehensive analysis underscores the significant potential of AI technology in enhancing the accuracy of CCA diagnoses, optimizing the formulation of personalized treatment plans, and predicting early recurrence risks. The current research hotspots primarily revolve around three directions: AI in the diagnosis and classification of CCA, AI in the preoperative assessment of cancer metastasis risk in CCA, and AI in the prediction of postoperative recurrence in CCA. This study highlights the pivotal role of AI technology in driving the treatment of CCA towards more personalized and precise directions, emphasizing the complementarity and interdependence among different AI applications, and pointing to the broad prospects for AI in the future healthcare domain.

## Limitations

This study also has certain limitations. Firstly, bibliometric analysis often relies on literature from the WoSCC database, which may lead to the omission of important research or literature outside this database, affecting the completeness and accuracy of the analysis. Secondly, the rapid development of Artificial Intelligence technology, especially in the field of deep learning, with new research findings continuously emerging, poses challenges to tracking its application in specific medical fields, particularly in conducting long-term trend analyses. Thirdly, bibliometric analysis generally cannot deeply assess the quality of individual studies, meaning that high-quality and low-quality research may be treated equally, impacting the accuracy of the comprehensive analysis results.
