# Supplementary material for: Research trends and hotspots evolution of artificial intelligence for cholangiocarcinoma over the past 10 years: a bibliometric analysis
Source: Front Oncol. 2025 Feb 13;14:1454411. doi: 10.3389/fonc.2024.1454411 (PMC11865243; doi:10.3389/fonc.2024.1454411)
Supplement: Supplementary file 1 [file Table1.docx]

**Supplementary Table1. The Topic Search Query**

**Web of Science Core Collection <January 1 2014 to December 31, 2023>**

| **Set** | **Results** | **Search Query** |
| --- | --- | --- |
| #1 | 17,150 | TS=("Cholangiocarcinoma" OR "Bile Duct Cancer" OR "Intrahepatic Cholangiocarcinoma" OR "Extrahepatic Cholangiocarcinoma" OR "Biliary Tract Cancer" OR "Biliary Cancer" OR "Klatskin Tumor" OR "Hilar Cholangiocarcinoma" OR "Bile Duct Neoplasms") |
| #2 | 1,797,795 | TS=("Artificial Intelligence" OR "AI" OR "Machine Learning" OR "ML" OR "Deep Learning" OR "Neural Networks" OR "Supervised Learning" OR "Unsupervised Learning" OR "Reinforcement Learning" OR "Natural Language Processing" OR "NLP" OR "Computer Vision" OR "Pattern Recognition" OR "Predictive Analytics" OR "Algorithm" OR "Cognitive Computing") |
| #3 | 834 | #1 AND #2 |
| #4 | 736 | #3 AND Article (Document Types) AND English (Languages) |

| **Supplementary Table 2** ┃Top 10 Frequency and Centrality of Keywords Related to Artificial Intelligence for Cholangiocarcinoma. | | | | | |
| --- | --- | --- | --- | --- | --- |
| **Rank** | **Frequency** | **Keywords** | **Rank** | **Centrality** | **Keywords** |
| 1 | 141 | intrahepatic cholangiocarcinoma | 1 | 0.12 | intrahepatic cholangiocarcinoma |
| 2 | 105 | hepatocellular carcinoma | 2 | 0.12 | expression |
| 3 | 99 | cancer | 3 | 0.11 | diagnosis |
| 4 | 97 | diagnosis | 4 | 0.1 | cancer |
| 5 | 92 | survival | 5 | 0.1 | risk |
| 6 | 91 | resection | 6 | 0.09 | cholangiocarcinoma |
| 7 | 90 | management | 7 | 0.09 | biliary tract cancer |
| 8 | 85 | cholangiocarcinoma | 8 | 0.08 | hepatectomy |
| 9 | 66 | hilar cholangiocarcinoma | 9 | 0.08 | cells |
| 10 | 58 | carcinoma | 10 | 0.07 | survival |

| **Supplementary Table 3** ┃Top 10 Authors Related to Artificial Intelligence for Cholangiocarcinoma. | | | | |
| --- | --- | --- | --- | --- |
| **Rank** | **Author** | **Frequency** | **Year** | **Country** |
| 1 | Aldrighetti, Luca | 16 | 2015 | Italy |
| 2 | Pawlik, Timothy M | 15 | 2015 | USA |
| 3 | Shen, Feng | 11 | 2018 | Peoples R China |
| 4 | Maithel, Shishir K | 10 | 2017 | USA |
| 5 | Guglielmi, Alfredo | 9 | 2020 | Italy |
| 6 | Bauer, Todd W | 9 | 2015 | USA |
| 7 | Poultsides, George A | 8 | 2019 | USA |
| 8 | Weiss, Matthew | 8 | 2019 | USA |
| 9 | Alexandrescu, Sorin | 8 | 2019 | Romania |
| 10 | Pulitano, Carlo | 8 | 2019 | Australia |

| **Supplementary Table 4** ┃Top 10 Frequency and Centrality of Countries Related to Artificial Intelligence for Cholangiocarcinoma. | | | | | |
| --- | --- | --- | --- | --- | --- |
| **Rank** | **Frequency** | **Countries** | **Rank** | **Centrality** | **Countries** |
| 1 | 292 | PEOPLES R CHINA | 1 | 0.37 | FRANCE |
| 2 | 128 | JAPAN | 2 | 0.15 | PEOPLES R CHINA |
| 3 | 116 | USA | 3 | 0.15 | GERMANY |
| 4 | 57 | THAILAND | 4 | 0.13 | EGYPT |
| 5 | 55 | GERMANY | 5 | 0.13 | ARGENTINA |
| 6 | 52 | ITALY | 6 | 0.12 | SOUTH KOREA |
| 7 | 48 | SOUTH KOREA | 7 | 0.12 | ENGLAND |
| 8 | 24 | FRANCE | 8 | 0.11 | SWITZERLAND |
| 9 | 22 | ENGLAND | 9 | 0.1 | USA |
| 10 | 20 | CANADA | 10 | 0.1 | ITALY |

| **Supplementary Table 5** ┃Top 10 Publications of Institutions Related to Artificial Intelligence for Cholangiocarcinoma. | | | |
| --- | --- | --- | --- |
| **Rank** | **Frequency** | **Year** | **Institutions** |
| 1 | 37 | 2014 | Khon Kaen University |
| 2 | 34 | 2014 | Naval Medical University |
| 3 | 29 | 2017 | Zhejiang University |
| 4 | 23 | 2014 | Sun Yat Sen University |
| 5 | 22 | 2016 | Sichuan University |
| 6 | 22 | 2017 | Stanford University |
| 7 | 21 | 2017 | Chinese Academy of Medical Sciences - Peking Union Medical College |
| 8 | 21 | 2017 | Fudan University |
| 9 | 17 | 2015 | Assistance Publique Hopitaux Paris (APHP) |
| 10 | 17 | 2015 | Johns Hopkins University |

| **Supplementary Table 6** ┃Top 10 Frequency of Cited Journals Related to Artificial Intelligence for Cholangiocarcinoma. | | | |
| --- | --- | --- | --- |
| **Rank** | **Frequency** | **Year** | **Cited Journals** |
| 1 | 292 | 2014 | HEPATOLOGY |
| 2 | 285 | 2014 | ANN SURG |
| 3 | 274 | 2014 | ANN SURG ONCOL |
| 4 | 252 | 2014 | J HEPATOL |
| 5 | 208 | 2014 | HPB |
| 6 | 192 | 2014 | SURGERY |
| 7 | 190 | 2014 | J GASTROINTEST SURG |
| 8 | 187 | 2014 | J CLIN ONCOL |
| 9 | 186 | 2014 | WORLD J GASTROENTERO |
| 10 | 180 | 2014 | WORLD J SURG |
